# Hospital Admissions, Transfers and Costs of Guillain-Barré Syndrome

**DOI:** 10.1371/journal.pone.0143837

**Published:** 2016-02-09

**Authors:** Nikki van Leeuwen, Hester F. Lingsma, Ann M. Vanrolleghem, Miriam C. J. M. Sturkenboom, Pieter A. van Doorn, Ewout W. Steyerberg, Bart C. Jacobs

**Affiliations:** 1 Centre for Medical Decision Making, Department of Public Health, Erasmus Medical Centre, PO Box 2040, 3000 CA, Rotterdam, The Netherlands; 2 Department of Medical Informatics, Erasmus Medical Centre, Rotterdam, The Netherlands; 3 Department of Neurology, Erasmus Medical Centre, Rotterdam, The Netherlands; 4 Department of Immunology, Erasmus Medical Centre, Rotterdam, The Netherlands; Friedrich-Alexander University Erlangen, GERMANY

## Abstract

**Background:**

Guillain-Barré syndrome (GBS) has a highly variable clinical course, leading to frequent transfers within and between hospitals and high associated costs. We defined the current admissions, transfers and costs in relation to disease severity of GBS.

**Methods:**

Dutch neurologists were requested to report patients diagnosed with GBS between November 2009 and November 2010. Information regarding clinical course and transfers was obtained via neurologists and general practitioners.

**Results:**

87 GBS patients were included with maximal GBS disability score of 1 or 2 (28%), 3 or 4 (53%), 5 (18%) and 6 (1%). Four mildly affected GBS patients were not hospital admitted. Of the 83 hospitalized patients 68 (82%) were initially admitted at a neurology department, 4 (5%) at an ICU, 4 (5%) at pediatrics, 4 (5%) at pediatrics neurology and 3 (4%) at internal medicine. Median hospital stay was 17 days (IQR 11–26 days, absolute range 1–133 days). Transfers between departments or hospitals occurred in 33 (40%) patients and 25 (30%) were transferred 2 times or more. From a cost-effectiveness perspective 21 (25%) of the admissions was suboptimal. Median costs for hospital admission of GBS patients were 15,060 Euro (IQR 11,226–23,683). Maximal GBS disability score was significantly correlated with total length of stay, number of transfers, ICU admission and costs.

**Conclusions:**

Hospital admissions for GBS patients are highly heterogeneous, with frequent transfers and higher costs for those with more severe disease. Future research should aim to develop prediction models to early identify the most cost-effective allocation in individual patients.

## Background

Guillain-Barré syndrome (GBS) is a life-threatening immune-mediated polyradiculoneuropathy [1–2] which requires early diagnosis and hospital admission for accurate monitoring, treatment and supportive care.

GBS was initially treated with plasma exchange (PE) in specialized centers, but since the introduction of intravenous immunoglobulin (IVIg) in 1992, care for GBS patients was highly decentralized.[3–5] GBS is a heterogeneous disorder regarding clinical presentation and course and patients highly differ with respect to the required duration and intensity of hospital care.[6] Diagnosis may be delayed, especially in patients with atypical clinical presentation, including pain[7], and in young children.[8] After admission, patients may rapidly progress and require intensive monitoring or long-term ventilator support at an Intensive Care Unit (ICU), which may not be available in smaller hospitals. Diagnostic delay and unexpected deteriorations in 8% to 16% of the patients [4,9], may cause more (acute) transfers between wards and ICUs or between local and academic hospitals. Previous studies showed that transfers of critically ill patients and emergency intubations in general result in longer stay at the ICU[10], and have a negative impact on patient and public health.[10,11]

Currently it is unknown in which departments and hospitals GBS patients are admitted, how often they are transferred, and what the associated costs are. In this study we aim to evaluate the current practice of hospital admissions, transfers and costs in relation to severity of disease, with the ultimate aim to provide optimal, cost-effective care for GBS patients.

## Methods

### Data collection and patient population

Data from the Pandemic Influenza & Vaccination (PIV) study were used, which was originally designed to investigate the relation between GBS and the pandemic influenza A (H1N1) virus.[12] Neurologists from all Dutch hospitals were requested to report patients diagnosed with GBS between November 2009 and November 2010. All the neurologists reported the GBS patients on a voluntary basis. Consequently, information regarding diagnostic features, clinical course and transfers was obtained via neurologists, general practitioners and discharge letters from the hospital that was specifically approved by the Medical Ethical Committee of the Erasmus Medical Center in Rotterdam. Written informed consent was not given by participants for their clinical records to be used in this study. Patient information was anonymized and de-identified prior to analysis.

### Definitions

All patients included fulfilled the diagnostic criteria for GBS from the Brighton Collaboration.[13] Clinical severity was defined by the GBS disability score at nadir (0 = healthy, 1 = minor symptoms, 2 = able to walk 10m unassisted but unable to run, 3 = able to walk over 10m open space with help, 4 = bedridden or chair bound, 5 = requiring ventilation for at least a part of the day, 6 = dead). For each patient, the number of transfers was determined. Two transfers equal three beds (e.g. patient admitted at a neurology department, transferred to an ICU and transferred back to the same neurology department). We counted both transfers between hospitals and between departments within a single hospital. Hospitals were divided in three categories; local, top clinical and academic centres. Top clinical centres are non-academic “high cure” centres, which have a high level ICU facility were prolonged mechanical ventilation is possible.[14]

### Specific Patterns of Admission and Transfer

Five specific transfer patterns were identified which might be suboptimal in terms of cost-effectiveness:

1. In adults a first admission to another department than neurology or ICU, as this may indicate misdiagnosis.
2. In children ≤ 18 year first admission to another department than pediatrics neurology as children with GBS may be misdiagnosed and require specialized neurological care.[8]
3. Relatively mildly affected patients (maximal GBS disability score ≤ 3) admitted to an academic centre or an ICU, as this might implicate unnecessary high costs.
4. Inter-hospital transfers from local to academic centre in the first two days of hospital admission, as such a rapid deterioration might have been anticipated on with direct admission to an academic centre.
5. Mechanical ventilation at the ICU in a local smaller centre, as GBS patients may require mechanical ventilation for extensive periods of time and require specialized care in larger centres (at least level 2 ICU in The Netherlands).

### Costs of GBS hospital admission

We included costs of admission days in general and academic hospitals, admission days at ICU, treatment with IVIg and transfers between hospitals. Costs consist of cost prices and volumes. Cost prices are the costs of one single cost unit, e.g. one admissions day. The cost prices were obtained from standardized cost-data in “Manual for cost research”.[15] Costs for medical doctors, ward doctors, nurses, other staff members, equipment, medical devices, food, standard medicines, housing and overhead costs were included in the cost prices per one admission day at the intensive care unit. The costs for mechanical ventilation are not charged separate from the costs for one admission day at the intensive care unit, since the costs for the equipment and extra monitoring of the patient are already included in the cost price for an admission day at the intensive care unit. Volumes are the number of a cost unit, thus the number of admission days. We did not have specific information on the type of treatment in all individual patients with GBS. In The Netherlands the first choice treatment according to the national CBO guideline for GBS is IVIg, which is also available in all centers. According to this guideline treatment is indicated in patients with GBS disability score ≥ 3) or who are transferred to the ICU and in this study costs for treatment with one course of IVIg was allocated. Consequently, by multiplying cost prices with volumes the total costs per patient were calculated. Mean and median costs with interquartile ranges (IQR) in Euros were assessed for the total study population and for each maximal GBS disability score subgroup. To assess which patient characteristics mainly determine costs, a linear regression model was fitted with age and maximal GBS disability score as independent variables and costs as dependent variable. The total costs of all GBS hospital admissions in The Netherlands per year were determined by multiplying the incidence of GBS per year in the total Dutch population by the median hospital costs.

### Statistical analyses

Patient characteristics and hospital admissions were described as medians with IQRs and absolute ranges, or as frequencies. Spearman correlation coefficients (SCC) and corresponding p-values were calculated for correlations between maximal GBS disability score and total length of stay, frequency of transfers, ICU admission and days to first transfer. Similarly, correlation coefficients were calculated for maximal GBS disability score with days between hospital admission and transfer to the ICU, length of stay at ICU and total length of stay in patients admitted to an ICU during hospital stay.

All analyses were performed with SPSS 20.0 (SPSS Inc, Chicago, Illinois), figures were made with Graphpad Prism 6.01 (Graphpad Software Inc) and R statistical software 2.15.3 (R Foundation for Statistical Computation, Vienna, Austria).

## Results

### Patient population and characteristics

The study population consisted of 87 GBS patients from a representative combination of 41 different hospitals in The Netherlands (13% academic, 33% top clinical and 58% local centres in our cohort compared to 9%, 31% and 60% in The Netherlands). The maximal GBS disability scores were: 1 or 2 (28%), 3 or 4 (53%), 5 (18%) and 6 (1%) (Table 1) and was representative for the general population of GBS patients as described in a previous Dutch observational GBS study.[7] Four (5%) patients had a relatively mild variant of GBS, not reaching a GBS disability score >3. They were not hospitalized and excluded from further analyses. The hospitalized patients had a median age of 49 (IQR 30–63), with 11 (13%) children (≤ 18 years old) and 48 (59%) males.

**Table 1:** Characteristics of 83 hospitalized patients with GBS.

Characteristics		Missing (%)
**Age, median (IQR)**	49 (30–64)	0 (0)
**Sex, male (%)**	49 (56)	1 (1)
Severity at nadir (maximal GBS disability score)*		0 (0)
**1 (%)**	4 (5)	
**2 (%)**	16 (19)	
**3 (%)**	25 (30)	
**4 (%)**	21 (25)	
**5 (%)**	16 (19)	
**6 (%)**	1 (1)	
**Preceding diarrhoea (%)**	13 (26)	33 (40)
**Facial and/or bulbar weakness (%)**	30 (36)	4 (5)
**Days between onset weakness and admission, median (IQR)**	2 (0–5)	6 (8)
**Length of stay in hospital**		2 (2)
**median (IQR)**	17 (11–26)	
**absolute range**	1–133	
**1**^**st**^ **hospital**		0 (0)
**Academic centre (%)**	12 (15)	
**Top clinical centre (%)**	33 (40)	
**Local centre (%)**	38 (46)	
Departments during hospital stay**		
**Neurology (%)**	74 (89)	
**ICU (%)**	26 (31)	
**Medium Care/ Neurology- ICU (%)**	4 (5)	
**Internal Medicine (%)**	3 (4)	
**Paediatric Neurology (%)**	6 (7)	
**Paediatrics (%)**	4 (5)	
**Transfers during hospital stay**		0 (0)
**0 (%)**	50 (60)	
**1 (%)**	7 (8)	
**2 (%)**	18 (22)	
**3 (%)**	6 (7)	
**4 (%)**	2 (2)	
**Days between 1**^**st**^ **and 2**^**nd**^ bed, median (IQR)***	2 (1–4)	5 (15)
**ICU admission**		
**mechanical ventilation (%)**	17 (65)	
**Discharge direction Home**	41 (49)	2 (2)
** Rehabilitation centre**	37 (45)	
** Nursing home**	3 (4)	

Data are presented as numbers (percentages) or medians (interquartile ranges), excluding patients with missing data.

* 1 = minor symptoms, 2 = able to walk 10m unassisted but unable to run, 3 = able to walk over 10m open space with help, 4 = bedridden or chair bound, 5 = needs ventilation for at least a part of the day, 6 = dead.

** These figures indicate in which departments the GBS patients were admitted at some time point during hospital stay. A proportion of patients was admitted at various departments, explaining the total number exceeds 83.

*** Calculated for patients with at least one transfer (n = 33).

### Hospital admissions

**Fig 1:**
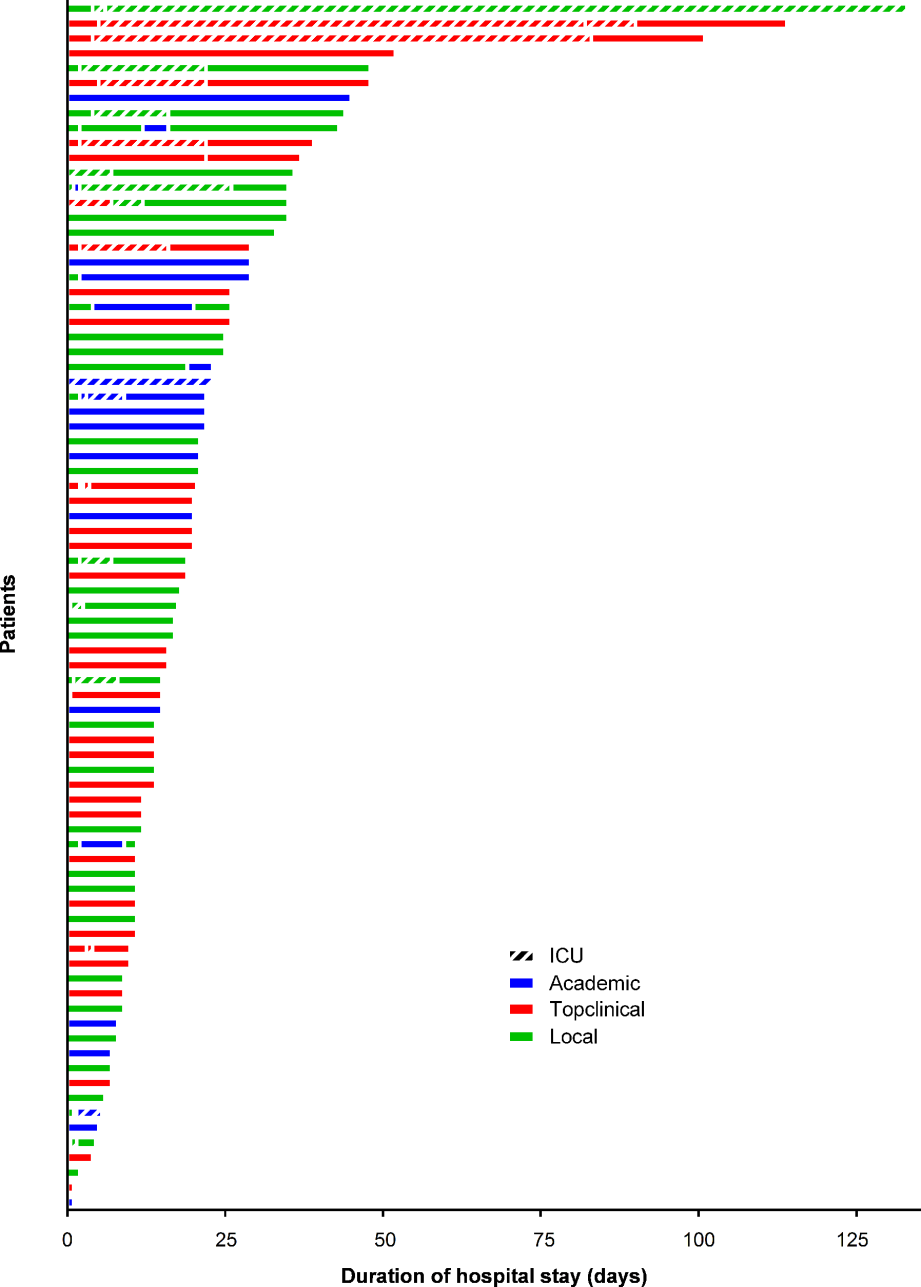
Overview hospital stay of 87 GBS patients. 7 patients not included in figure since limited data were available on exact days of admission and department(s) of admission. Hatched bars are ICU admissions, non-hatched bars are admissions in any other department.

Of 83 hospitalized patients, 12 (15%) were initially referred to an academic centre, 33 (40%) to a top clinical centre and 38 (46%) to a local centre. The patients were initially referred to various departments: 68 (82%) to a neurology department, 4 (5%) to an ICU, 3 (4%) to internal medicine, 4 (5%) to pediatrics, and 4 (5%) to pediatric neurology. The median hospital stay was 17 days (IQR 11–26 days; absolute range 1–133 days). A higher maximal GBS disability score was significantly correlated with a longer total length of stay (SCC 0.59, p < 0.001) (Table 2). Of the 83 admitted patients, 33 (40%) had at least one transfer to another department or hospital, and more than 50% of patients were transferred within 2 days after admission. Moreover, 26 (31%) patients were transferred 2 times or more of which 2 (2%) were transferred 4 times. A higher maximal GBS disability score was significantly correlated with more transfers (SCC 0.62, p < 0.001). One patient had died in the hospital. More detailed information regarding the hospital admission is presented in Table 1. The course of hospital admission for all patients is presented in Fig 1.

**Table 2:** Spearman correlations with maximal GBS disability score.

**Total population (n = 83)**	Correlation coefficient	P-value
Total length of hospital stay	0.59	<0.001
Frequency of transfers	0.62	<0.001
ICU admission*	0.67	<0.001
Days to first transfer**	0.15	0.44
ICU admissions (n = 26)***	Correlation coefficient	P-value
Days between hospital admission and transfer to ICU	0.20	0.38
Length of stay at ICU	0.46	0.03
Total length of stay	0.37	0.08

* This correlation coefficient is based on the total GBS cohort. The correlation coefficient for patients with a maximal GBS disability score ≤ 4 is 0.28, p = 0.03.

** This correlation coefficient is based on GBS patients with at least one transfer (n = 33).

***All correlation coefficients below are calculated only for patients admitted at an ICU (n = 26).

### ICU admissions

26 (31%) patients stayed at an ICU at some time during follow-up, of which 17 (65%) were ventilated. In patients with a GBS disability score of 4 or lower (i.e. not by definition admitted to the ICU) a higher maximal GBS disability score was significantly correlated to ICU admission (SCC 0.28, p = 0.025) (Table 2). Median time between onset of weakness and admission to the ICU was 4 days (IQR 2–7 days; absolute range 1–14 days). Median length of stay at the ICU was 12 days (IQR 2–20 days). A higher maximal GBS disability score was significantly correlated to a longer stay at the ICU (SCC 0.46, p = 0.03). Median duration of ICU admission was 4 days (IQR 1–10 days) for patients with a maximal GBS disability score of 4 and 17 days (IQR 10–24 days) for patients with a maximal GBS disability score of 5. Main (documented) reasons for transfer to an ICU were (risk for) mechanical ventilation or short-term admission for observation.

### Patients transferred between different types of hospitals

Nine (11%) patients were transferred between different types of centres, including 7 patients from a local centre to an academic centre (median time of transfer after admission was 2 days; range 1–19 days). Two patients were transferred from a top clinical centre to an academic centre (after 1 day) or local centre (after 7 days). No patients were transferred from a local to a top clinical centre, and no patients initially admitted to an academic centre were transferred to another type of hospital. 78% of the inter-hospital transfers occurred in the first week of admission and in 56% of the patients within two days. Five (56%) patients were children (≤ 18 years) and 3 (33%) patients had a maximal GBS disability score 5.

### Specific patterns of admission and transfer

In 21 (25%) patients, the admission and transfers might be classified as suboptimal from a cost-effectiveness perspective.

1. Three (4%) patients were initially admitted to an internal medicine department.
2. Four (5%) children were initially admitted to a general pediatric department, and transferred to pediatric neurology department or ICU. Three of them were transferred from a local to an academic centre.
3. Six (7%) relatively mildly affected patients (maximal GBS disability score ≤ 3) were admitted to an academic centre (3; 4%) or ICU (3; 4%).
4. Four (5%) patients were transferred within 2 days of admission from a local to an academic centre.
5. Seven (8%) patients were mechanically ventilated in a local centre.

### Costs of GBS hospital admissions

Seven patients were excluded from cost analyses because of lack of data. Median costs of the remaining 80 patients were 15,060 Euro (IQR 11,226–23,683 Euro), with an absolute range of 575–208,018 Euro. These costs were composed of admission days in a general hospital (435 Euro per day), academic hospital (575 Euro per day), or ICU (2183 Euro per day), frequency of inter-hospital transfers (262 Euro per transfer) and treatment with one course of IVIg (8,100 Euro per course). The estimated total costs for all GBS hospital admissions in the Netherlands per year were 4,832,000 Euro (estimated frequency in total Dutch population of 200 patients multiplied by the median hospital costs of 24,160 Euro).

**Fig 2:**
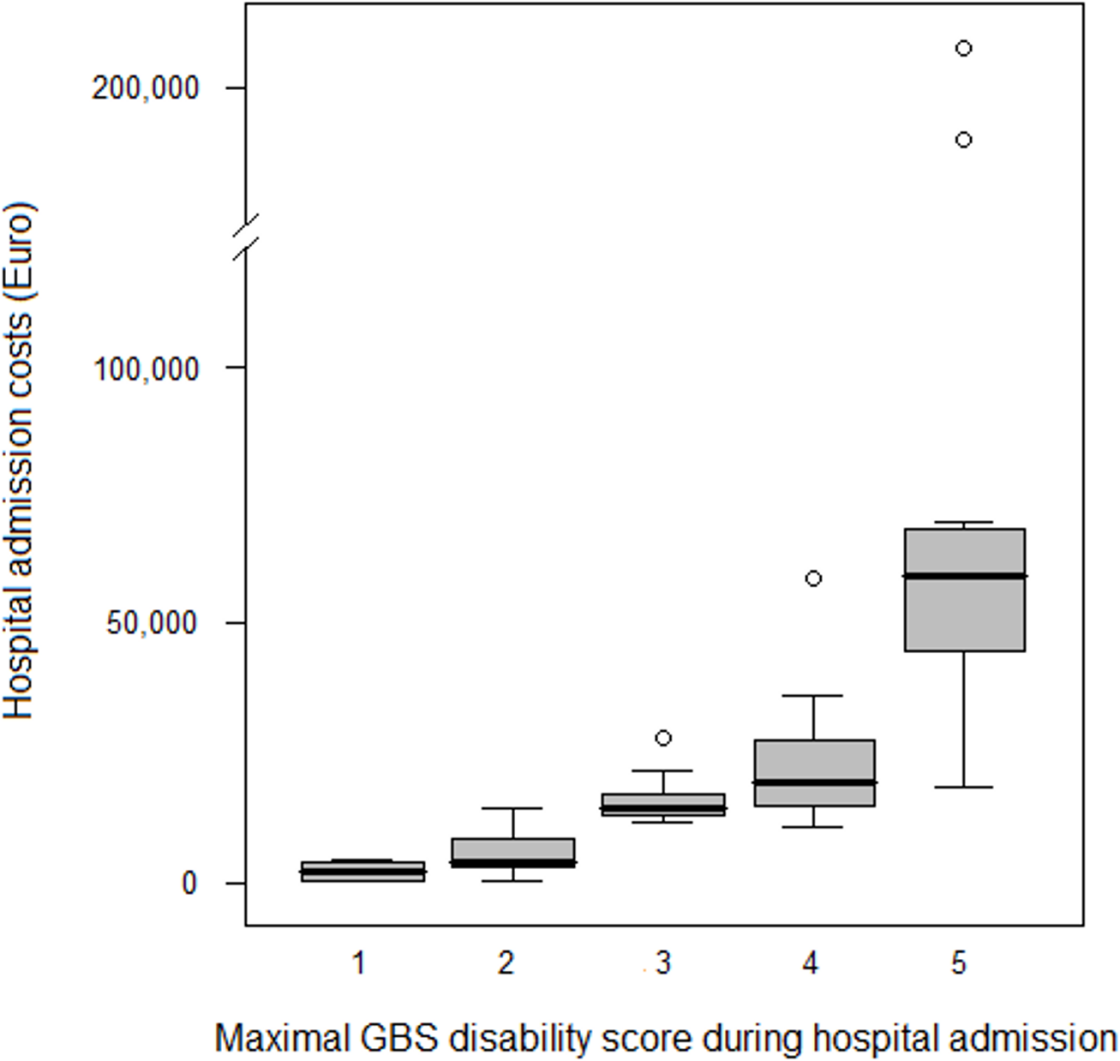
Interquartile ranges (grey boxes), 95% confidence intervals (whiskers) and median (dark lines in middle of the boxes) of costs of hospital admission for different maximal GBS disability scores. Excluded was one patient who died. Circles are (extreme) outliers. Maximal GBS disability score during hospital admission: 1 = minor symptoms, 2 = able to walk 10m unassisted but unable to run, 3 = able to walk over 10m open space with help, 4 = bedridden or chair bound, 5 = needs ventilation for at least a part of the day.

Median costs were highly associated with disease severity (expressed as maximal GSB disability score), ranging from 2,428 Euro (IQR 796–3,806 Euro) for patients with a score of 1, to 59,167 Euro (IQR 45,031–68,369 Euro) for patients with a score of 5 (Table 3). The correlation between GBS disability score and costs was observed in both children and adults (Fig 2).

**Table 3:** (Minimum) Costs* of hospital admission in GBS patients.**

Maximal GBS disability score	N	Mean costs in Euros	Median costs in Euros (IQR)
1	4	2,428	2,175 (796–3,806)
2	16	5,558	4,258 (3,045–8,644)
3	23	15,866	14,625 (13,320–17,018)
4	20	22,715	19,296 (15,219–26,384)
5	12	75,066	59,167 (45,031–68,369)
6	1	17,529	17,529
**Total**	**76**	**24,160**	**15,060 (11,226–23,683)**

*These costs include costs for nursing days, treatment and transfers. Costs of diagnostic tests, physiotherapy and mechanical ventilation were not included in our calculations.

**Excluded were 7 patients from the cost analyses since limited data were available on exact days of admission and department(s) of admission.

The beta for the effect of maximal GBS disability score on costs (adjusted for age) was 16,442 (95% CI 10,939–21,945). This means, for example, that the costs for a patient with a maximal GBS disability score of 4 are is on average 16,442 Euro higher than those for a patient with a maximal GBS disability score of 3.

## Discussion

In this study the current practice of hospital admissions of patients with GBS was evaluated in terms of location, duration, transfers and costs, in a representative cohort of GBS patients in The Netherlands. Transfers within and between hospitals were frequent: 40% of the patients were transferred at least one time and half of them were transferred within 2 days of admission. Moreover, in 25% the admission may have been suboptimal form a cost-effectiveness perspective, including admission to other than (paediatric) neurology departments or ICUs, admission of mildly affected patients to ICUs and transfers shortly after the initial admission. The related costs were highly variable between patients and mainly associated with the severity of disease. These findings may suggest that the care of GBS patients in The Netherlands can be improved by developing more cost-effective referral strategies based on early diagnosis and prediction of clinical course and outcome.

### Strengths and limitations

Only very few studies have described the practice of current hospital admission of patients with GBS. Most studies on the clinical course of GBS are based on data from therapeutic trials, which may be biased to severe cases. Although reporting of GBS cases by the neurologists was voluntary in this study, we had a representative cohort of GBS patients. In the Netherlands, all care for all patients with GBS is primarily coordinated by neurologists. Therefor it is highly unlikely that GBS cases were missed because treatment was coordinated at another department. The types of hospital (academic, top clinical and local) were similarly distributed as the total number of hospitals in the Netherlands. The distribution of age, disease severity at nadir, proportion of ventilated patients was similar to previous studies on GBS patients in The Netherlands. Previous studies were performed in the United States, which has a different health care system than European countries, and focused largely on indirect costs.[16] Other studies only measured the costs of specific treatments for GBS[17–19] or analyzed costs of a specific subgroup of GBS.[20] In the current study we aimed to determine the current costs of hospital admissions across the full spectrum of this heterogeneous disorder.

### Optimal and cost effective care for GBS

GBS is a complex disorder for cost-effective care because of the various stages in the clinical course and diversity in clinical course between patients. The complexity is reflected in the high frequency of transfers between departments and hospitals, especially shortly after initial admission. Patients initially admitted at the internal medicine department may result in delayed specialized treatment and monitoring, and an extra transfer. From a costs point of view, ideally mildly affected patients are admitted to a local or top clinical centre with good general care for GBS but relatively low costs. More severely affected patients with a higher chance of respiratory failure and complications may benefit from admission in a top-clinical or academic centre. Four patients were transferred from a local to an academic centre within two days of admission and ideally these patients would have been admitted directly to a specialized centre. Adequate assessment of prognosis could aid decision making at the time of admission. Prognostic models have been developed to support this assessment, including the externally validated modified Erasmus GBS Outcome Score (mEGOS) to predict disability outcome in GBS patients at the time of admission.[21]

Seven patients were initially mechanically ventilated in a local centre, which could have been prevented when earlier transferred to a top clinical or academic centre. The Erasmus GBS Respiratory Insufficiency Score (ERGIS) [22] was developed to predict respiratory insufficiency at time of admission. When a patient has a high chance of respiratory insufficiency, careful monitoring can potentially avoid an unexpected emergency intubation and acute transfer to the ICU. The ERGIS could help clinicians to decide to admit or transfer a patient to an academic centre before the critical stage of disease. Direct admission to a top clinical or academic is preferred above transfer since inter-hospital transfers have negative impact on patient outcome.[10,11] ERGIS could also help avoiding unnecessary ICU admissions of mild GBS patients to save costs. In this study, 9 of the ICU admitted patients had no need for mechanical ventilation. We cannot exclude the possibility that these patients were admitted to an ICU because of autonomic dysfunction, although this sole indication for admission to an ICU may be relatively rare.

### Children with GBS

Almost half of the children in this cohort were initially admitted to a general pediatrics department. Considering the challenging neurologic examination, monitoring and treatment of children with GBS, they should preferably be seen by a pediatric neurologist and be admitted to a centre with a pediatric ICU. All children initially admitted to a pediatric department were later transferred to a pediatric neurology department or ICU. This referral pattern may indicate a delay in diagnosis of GBS in young children compared to adults or problems with monitoring children during the progressive state.[8] In one child, admitted to a pediatric department in a local centre, the delayed diagnosis and insufficient monitoring resulted in death due to hypoxia after emergency intubation.[8,23]

### Costs of GBS hospital admission

We found that the costs of hospitals admission in GBS are highly variable and mainly depend on maximal GBS disability score. These are the minimal costs of GBS hospital admissions, since costs of diagnostic tests, physiotherapy and mechanical ventilation were not included. Moreover, one course with IVIg for each patient was assigned, although some patients may have received more (or no) course(s) with IVIg due to treatment related fluctuations or received other treatment like PE.

Length of stay is the main driver for high costs in GBS hospital admission, especially (long) admission to an ICU. This also explains the strong correlation between costs and GBS disability score. Compared to other costs during hospital admission, costs for inter hospital transfers are relatively low. Also a course with IVIg (8,100 Euro), although considered to be an expensive treatment, has relatively low costs compared to ICU admission (2,183 Euro per day).

## Conclusion

In conclusion, substantial heterogeneity in admission and transfer patters of GBS patients and associated costs was found. As this study lacks outcome data, no definite conclusions can be drawn, but we suggested several possibilities for improving to cost-effectiveness of care for GBS patients. Future research should focus on identifying subgroups of patients who benefit most from specialized care in an academic centre, e.g. based on prognostic models, and subsequently on developing admission guidelines to provide optimal, cost-effective care for GBS patients.

## Supporting Information

S1 Table Transfers between different types of hospitals (n = 9). (DOCX)
