# Supplementary material for: Hospital Admissions, Transfers and Costs of Guillain-Barré Syndrome
Source: PLoS One. 2016 Feb 9;11(2):e0143837. doi: 10.1371/journal.pone.0143837 (PMC4747559; doi:10.1371/journal.pone.0143837)
Supplement: S1 Table — (DOCX) [file pone.0143837.s001.docx]

**S1 Table. Transfers between different types of hospitals (n=9).**

| **Age** | **Maximal GBS disability score** | **Length of stay in 1^st^ hospital (days)** | **Department admission 1^st^ hospital** | **Reason for transfer** | **Length of stay in 2^nd^ hospital** | **Department admission 2^nd^ hospital** | **Transferred back to 1^st^ hospital** | **Total length of stay in hospital(s) (days)** |
| --- | --- | --- | --- | --- | --- | --- | --- | --- |
| **From top clinical to academic centre (n=1)*** | | | | | | | | |
| 10 | 5 | 1 | Pediatrics Neurology | Unknown | 19 | ICU | Yes | N/A |
| **From top clinical to local centre (n=1)*** | | | | | | | | |
| 21 | 5 | 7 | ICU | Parents of patient live closer to the local centre on the other side of the county | 28 | ICU | No | 35 |
| **From local to academic centre (n=7)*** | | | | | | | | |
| 28 | 4 | 19 | Neurology | Unknown | 4 | Neurology | No | 23 |
| 5 | 4 | 2 | Pediatrics | Unknown | 27 | Pediatric Neurology | No | 29 |
| 11 | 3 | 4 | Pediatrics | Unknown | 16 | Pediatric Neurology | Yes | 26 |
| 36 | 4 | 2 | Neurology | Unknown | 7 | Neurology | Yes | 11 |
| 6 | 4 | 2 | Pediatrics | Unknown | 17 | Neurology | Yes | 22 |
| 4 | 6 | 1 | Pediatrics | Unknown | 4 | ICU | Patient died | 5 |
| 59 | 3 | 12 | Neurology | Risk for mechanical ventilation | 4 | Neurology | Yes | 43 |

** There were no transfers from an academic centre to another type of hospital and no transfers from a local centre to a top clinical centre.*
